# Persistence of a Stx-Encoding Bacteriophage in Minced Meat Investigated by Application of an Improved DNA Extraction Method and Digital Droplet PCR

**DOI:** 10.3389/fmicb.2020.581575

**Published:** 2021-01-20

**Authors:** B. Spilsberg, C. Sekse, Anne M. Urdahl, Live L. Nesse, Gro S. Johannessen

**Affiliations:** ^1^Section for Molecular Biology, Norwegian Veterinary Institute, Oslo, Norway; ^2^Section for Food Safety and Animal Health Research, Norwegian Veterinary Institute, Oslo, Norway

**Keywords:** stx-phages, STEC, persistence, DNA extraction, digital droplet PCR, minced meat

## Abstract

Shiga toxin-producing *Escherichia coli* (STEC) are important food-borne pathogens with Shiga toxins as the main virulence factor. Shiga toxins are encoded on Shiga toxin-encoding bacteriophages (Stx phages). Stx phages may exist as free bacteriophages in the environment or in foods or as prophages integrated into the host genome. From a food safety perspective, it is important to have knowledge on the survival and persistence of Stx phages in food products since these may integrate into the bacterial hosts through transduction if conditions are right. Here, we present the results from a study investigating the survival of a Stx phage in minced meat from beef stored at a suboptimal temperature (8°C) for food storage along with modifications and optimizations of the methods applied. Minced meat from beef was inoculated with known levels of a labeled Stx phage prior to storage. Phage filtrates were used for plaque assays and DNA extraction, followed by real-time PCR and digital droplet PCR (ddPCR). The results from the pilot study suggested that the initial DNA extraction protocol was not optimal, and several modifications were tested before a final protocol was defined. The final DNA extraction protocol comprised ultra-centrifugation of the entire phage filtrate for concentrating phages and two times phenol–chloroform extraction. The protocol was used for two spiking experiments. The DNA extraction protocol resulted in flexibility in the amount of DNA available for use in PCR analyses, ultimately increasing the sensitivity of the method used for quantification of phages in a sample. All three quantification methods employed (i.e., plaque assays, real-time PCR, and ddPCR) showed similar trends in the development of the phages during storage, where ddPCR has the benefit of giving absolute quantification of DNA copies in a simple experimental setup. The results indicate that the Stx phages persist and remain infective for at least 20 days under the storage conditions used in the present study. Stx phages in foods might represent a potential risk for humans. Although it can be speculated that transduction may take place at 8°C with subsequent forming of STEC, it can be expected to be a rare event. However, such an event may possibly take place under more optimal conditions, such as an increase in storage temperature of foods or in the gastrointestinal tract of humans.

## Introduction

Shiga toxin-producing *Escherichia coli* (STEC) are important food-borne pathogens and may cause serious infections in humans with Shiga toxins as the main virulence factor. The gastrointestinal tract of healthy ruminants is the main reservoir for STEC. Foodborne infections with STEC are most often reported to be associated with foods of animal origin (EFSA and ECDC, 2019). Shiga toxins are encoded on Shiga toxin-encoding bacteriophages (Stx phages), which are a large group of temperate lambdoid bacteriophages (Allison, 2007; Krüger and Lucchesi, 2015). They follow either lysogenic or lytic cycle. In the lysogenic state, Stx phages are integrated into the host genome and the expression of most phage genes, including *stx*, are repressed.

Stx phages may also exist as free phages in the environment or in foodstuff after lysis of Stx-containing bacteria (Martínez-Castillo and Muniesa, 2014), facilitating acquisition by *E. coli* or other *Enterobacteriaceae* by horizontal gene transfer through transduction, forming new variants of STEC or Shiga toxin-producing *Enterobacteriaceae* (Allison, 2007; Kaper and O'Brien, 2014). This may indicate that the presence of free bacteriophages, particularly when present in foodstuffs represent a hazard. Previous studies have shown that free Stx phages can be detected in environmental samples such as waste water and river water; fecal samples from humans, swine, and cattle; and foodstuffs such as beef and salad [reviewed in Martínez-Castillo and Muniesa (2014)]. Imamovic and Muniesa (2011) detected Stx phages in all the tested samples of minced beef and in approximately 2/3 of the samples of salads using real-time PCR for analysis. The average number of Stx phages (based on gene copy number) in minced beef was 1.6 × 10^3^/g. Around 50% of the samples tested also harbored infective Stx phages, as indicated by an increase in number of Stx phages after propagation with an appropriate bacterial host. It is thus important to consider the infectivity of Stx phages when discussing the risks associated with free phages in foodstuffs or other relevant matrices for food production.

Phages can, to various degrees, tolerate different temperatures, pH, and salinities and thus have different abilities to remain infective in the environment (Jończyk et al., 2011). Only a few studies have investigated the survival and persistence of Stx phages in different matrices such as feces and slurry, compost, and moisture-enhanced meat (Johannessen et al., 2005; Langsrud et al., 2014; Nyambe et al., 2016). The studies indicated that the phages were able to persist and remain infective in the matrices studied (Langsrud et al., 2014; Nyambe et al., 2016). In the compost model, however, described by Johannessen et al. (2005), both *E. coli* and the labeled Stx phage were effectively removed after being exposed to a temperature of >60°C for at least 5 days.

Nyambe et al. (2017) studied transduction of a Stx2 phage to different *E. coli* pathotypes in broth and different food matrices at an optimum growth temperature of 37°C of both donor and recipient. The results indicated that the Stx2 phage used could be transferred to different pathotypes of *E. coli* in food matrices. It is known that transduction is dependent on different environmental factors, such as pH, temperature, desiccation, and exposure to disinfectants as well as the Stx phage and the host (Imamovic et al., 2009; Rode et al., 2011). Studies by Imamovic et al. (2009) and Picozzi et al. (2012) have indicated that transduction does not occur at low temperatures (4°C), but may happen at higher temperatures such as 15 and 22°C with their experimental models, and it has been shown that transduction takes place in biofilms at 20°C (Solheim et al., 2013). It is well-known that foods might be stored under suboptimal temperatures (e.g., 8–10°C) for shorter (transport from shop and unpacking groceries) or longer periods of time (temperatures too high in the refrigerator) (Skuland et al., 2020). While several studies have dealt with the persistence and growth of STEC in minced meat under different storage temperatures (Duffy et al., 2014), less is known about Stx phages in minced meat and whether they persist and remain infective.

Studying the occurrence, persistence, and transduction of Stx phages in foodstuffs may represent a challenge as the methodologies applied have potential for improvement. The use of quantitative methods for estimation of the number of Stx phages are of importance for risk assessments. Quantitative methods are needed to estimate the total number of phages in a matrix, e.g., if the numbers of phages are reduced by a specific treatment. Traditionally, plaque assays, also called double-agar layer methods, have been used to enumerate Stx phages present in a sample (Imamovic et al., 2010). However, these are demanding as many plaque forming units (pfu) from Stx phages have proven to be hard to detect and enumerate correctly (Muniesa et al., 2004a,b; Islam et al., 2012). Different phage traits such as lysis time, virion morphology, and adsorption rate will affect the plaque properties such as plaque size (Gallet et al., 2011). Moreover, plaque assays might also depend upon the ability of the phages present in the matrix to infect the bacterial strain(s) used as host(s). All phages present that are able to infect the host strain will form plaques, and in order to identify Stx phages alone, plaque hybridization with a specific *stx* probe needs to be included. The background noise in such a method is high and accurate quantification is demanding. The number of phages and how they are extracted from the matrix will also have an impact as plaque assays might not be sensitive to low phage numbers.

Modern molecular methods, such as different PCR methods, offer alternatives to labor-intensive and often less-reliable plaque assays. Real-time PCR can, in contrast to classical PCR, quantify nucleic acid in samples as described for Stx phages (Imamovic et al., 2010; Rooks et al., 2010). Recently, digital PCR has become an alternative for quantitative PCR analysis. In digital PCR, quantification is done without an external standard curve and with less sensitivity to PCR inhibitors and can thus be a cost-efficient alternative to standard curve-based quantitative PCR (Hoshino and Inagaki, 2012; Zhao et al., 2016). When applying molecular methods, DNA extraction is an important, but often underrated part of the analysis, affecting sensitivity and specificity of the methods applied.

From a food safety perspective, it is necessary to evaluate the survival and persistence of free Stx phages in foods and the potential for phage-mediated transfer of *stx* genes. Here, we present the results from a study investigating the survival of a Stx phage in minced meat from beef stored at suboptimal temperature (8°C) along with optimization and further development of the methods applied.

## Materials and Methods

### Preparation of Inocula and Bacterial Strains

A Stx2-encoding bacteriophage, Φ731 (Δ*stx*_2_::*cat*), hereafter called Φ731, was used in the experiments. The Stx phage originate from STEC O103:H25 from a Norwegian HUS patient. The phage had a chloramphenicol acetyltransferase, *cat*, inserted into the *stx*_2_ gene (Serra-Moreno et al., 2006). *Escherichia coli* C600 was infected with Φ731 as described previously by Solheim et al. (2013) and Serra-Moreno et al. (2006). Phage filtrate was obtained from bacterial cultures of *E. coli* C600 [Φ731 (Δ*stx*_2_::*cat*)] grown in 100 ml Luria-Bertani (LB) broth and induced with Mitomycin C as described previously (Solheim et al., 2013). The overnight culture was centrifuged at 10,000 × *g* for 30 min, and the supernatant was sterile filtered using 0.22 μM low-protein binding membranes (Steritop 0.22 μm GP Millipore Express, Millipore). *Escherichia coli* C600 was used as host strain for plaque assays throughout the study.

### Experimental Design

#### Pilot Study

A pilot experiment was carried out using minced meat from beef. Pre-packed minced bovine meat with 14% fat and no added salt or water (400-g packages) were obtained from a local supermarket. The packages were inoculated separately with 100 μl phage filtrate containing ~3 × 10^7^ pfu through a silicone patch in order to maintain the modified atmosphere in the package (De Cesare et al., 2018). Negative control samples were inoculated with 100 μl phosphate-buffered saline (PBS). The samples were stored at 8°C, a suboptimal temperature for storage of minced meat, and samples were collected immediately after time of inoculation (T0) and after 1 and 3 days (T1 and T3). Five gram of minced meat was taken directly underneath the inoculation patch and diluted with 15 ml PBS, followed by quantification using plaque assays (see section Plaque Assays). DNA extraction was performed using a non-modified protocol [see section DNA Extraction and Supplementary Material 1 (1.1)], and real-time PCR (see section PCR Assays) was performed on the extracted phage DNA. The negative control samples were analyzed by plaque assays (see section Plaque Assays) and for the hygiene indicators *E. coli* and *Enterobacteriaceae* (see section Bacteriological Analyses).

#### Experiment 1

**Figure 1:**
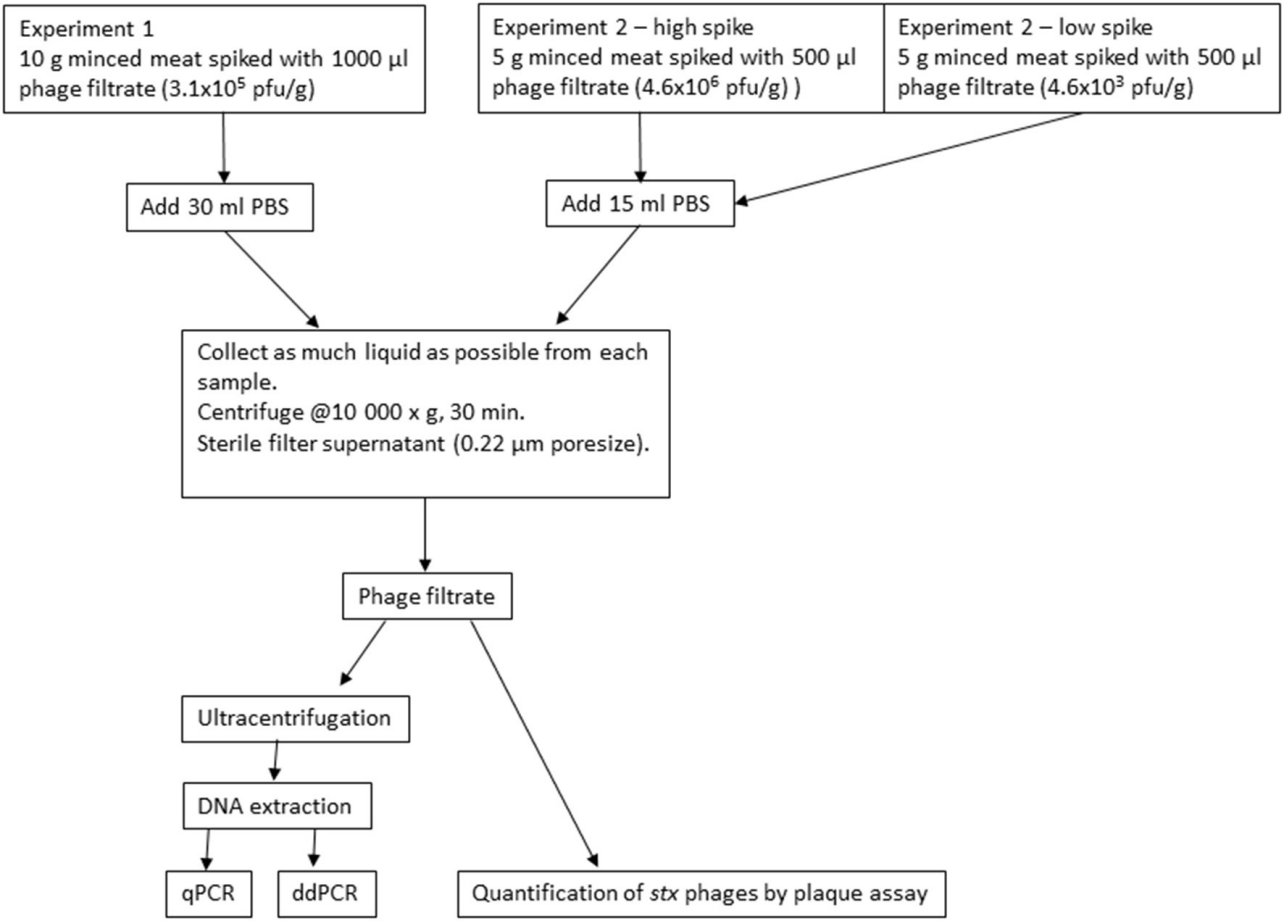
Flow diagram of the inoculation experiments with minced meat from beef.

Minced meat from beef with 14% fat and no added salt or water obtained at a local supermarket was used for experiment 1. Portions of 10 g were distributed into 50-ml tubes and stored at 4°C until inoculation with bacteriophage filtrate. The samples were inoculated separately with 3.1 × 10^5^ pfu/g Φ731 (1,000 μl) and stored at 8°C for up to 20 days. On the day of inoculation (T0) and 1, 3, 8, 10, and 20 days post inoculation (T1, T3, T8, T10, and T20), three randomly selected parallels were subjected to further analysis. DNA was extracted from the samples using the final modified protocol described in the section DNA Extraction and Supplementary Material 2. A flow diagram of the experiments is shown in Figure 1.

Negative control samples were prepared at the same time and stored under the same conditions as the inoculated samples. The control samples were analyzed on the day prior to inoculation (T0–1) and T10 (2 days after use-by date) for plaque assays (see section Plaque Assays) and selected bacteriological analyses (see section Bacteriological Analyses).

#### Experiment 2

In experiment 2, the experimental set-up was downscaled for practical reasons; however, the ratio between sample size and inoculum remained the same (Figure 1). Minced meat from beef with 5% fat and no added salt or water obtained from a local supermarket was used. Portions of 5 g were distributed into 50-ml tubes and inoculated with 500 μl of Φ731 filtrate. Two different concentrations of Φ731 filtrate were used in experiment 2. The high spike (4.6 × 10^6^ pfu/g) was ~1log higher than in experiment 1. The low spike (4.6 × 10^3^ pfu/g) was used to obtain a more realistic level of phages present in the samples as observed by Imamovic and Muniesa (2011). The samples were stored and analyzed as described for experiment 1 (see section Experiment 1). The negative control samples were analyzed on the day prior to inoculation (T0–1) and on T20 (1 day after use-by date).

### Preparation of Samples for DNA Extraction and Plaque Assays

At the selected sampling times, three random tubes with spiked minced meat were analyzed. Each meat sample was transferred to a Stomacher bag with filter (VWR® Blender Bag, lateral filter, 400 ml, sterile) and diluted with 30 or 15 ml of PBS in experiment 1 and 2, respectively, followed by homogenization in a Stomacher for 1 min. As much liquid as possible was collected from the samples, transferred to 50-ml tubes, and centrifuged at 10,000 × *g* for 30 min at 4°C (Multifuge X3R, Thermo Scientific). The supernatant was filtrated through a 0.22-μm filter (Steritop 0.22 μm, 150 ml, Millipore, Merck), and 1–2 ml was collected and used for plaque assays as described (see section Plaque Assays) for quantification of Φ731. The remaining supernatant was used for DNA extraction (see section DNA Extraction).

### Plaque Assays

Quantification of phages was performed by plaque assay where the number of plaques on a soft agar plate was enumerated based on visual inspection of each plate as described by Jofre and Muniesa (2020). Aliquots of 100 μl of 10 fold dilutions of phage filtrate, 900 μl of the host bacteria, grown to exponential phase (OD 0.3–0.5), and 100 μl 0.1M CaCl_2_ were mixed and incubated at 37.0 ± 1.0°C for 30 min prior to mixing with 0.7% LB soft agar (3 ml) and poured onto pre-warmed LB agar plates. The LB soft-agar plates were incubated at 37.0 ± 1.0°C overnight. Plaque assays were carried out to quantify the number of plaque forming units (pfu) at the beginning of each experiment and at each selected time point.

### Enrichment of Phages With Host

In addition to plaque assays, a phage/host-enrichment method was applied to assess the presence of infective phages with the aim of generating lysogens at T10 and T20 and T20 in experiments 1 and 2, respectively. Phage filtrate obtained from the inoculated minced meat samples, host (*E. coli* C600 grown to OD 0.3–0.5), and LB broth in a total volume of 5 ml were mixed and incubated at 37.0 ± 1.0°C overnight. After incubation, 10 and 100 μl from the enrichment was plated on LB plates with 25 mg/l chloramphenicol. The plates were incubated at 37.0 ± 1.0°C overnight and inspected for growth of typical *E. coli* colonies. A selection of presumptive lysogens with Φ731 was tested using the Φ731-specific real-time PCR assay described in section PCR Assays.

### DNA Extraction

#### DNA Extraction

A DNA extraction method based on two phage DNA extraction protocols (Summer, 2009; Jofre and Muniesa, 2020) was tested for the pilot project. In brief, 1 ml phage filtrate was treated with DNase I and RNase A, and phages were concentrated using PEG (Jofre and Muniesa, 2020). DNA was further purified using the Wizard DNA Clean-Up System (Promega) according to the manufacturer's protocol [see Supplementary Material 1 (1.1)]. After the pilot study, several steps in the initial protocol were modified and optimized (see all modifications tested in Supplementary Material 1). Key steps we decided to modify were concentration of phages using ultra-centrifugation and changing to phenol–chloroform extraction in the final DNA extraction protocol, which was used in experiments 1 and 2. The final detailed DNA extraction protocol is described in Supplementary Material 2. Briefly, at first, phages were concentrated using ultra-centrifugation of the phage filtrate from the samples at 100,000 × *g* for 2 h (Beckman, SW41 TI swinging bucket). The pellet was dissolved in SM buffer, and then 1 μl of DNase I (1 U/μl, Promega) and 1 μl of RNase A (20 mg/ml, Sigma-Aldrich) were added and incubated at 37°C for 1 h. Further, 67 μl Proteinase K buffer, and 5 μl Proteinase K (20 mg/ml, Qiagen) were added and incubated at 50°C for 1 h. Phenol–chloroform extractions (phenol/chloroform/isoamyl alcohol 25:24:1, Sigma-Aldrich) using Phase Lock Gel tubes (Quantabio) were performed two times before DNA was precipitated with 0.7 volume isopropanol and washed with 70% ethanol. The DNA pellet was air-dried and resuspended in 50 μl TE-buffer, pH 8.0. After DNA extraction, DNA concentration was measured using a Qubit (Qubit BR kit, Thermo Fisher).

#### PCR Inhibition Test

To test whether three times phenol–chloroform extraction resulted in DNA of higher purity than two times, DNA was extracted from unspiked minced meat using two and three times phenol–chloroform extraction [in triplicates, see Supplementary Material 1 (1.6)]. A PCR inhibition test to assess DNA purity was performed using the real-time PCR assay described in the next section (PCR Assays). From 0 to 9.5 μl of DNA extracted from unspiked minced meat were analyzed together with a constant amount of Φ731 phage DNA. The Φ731 DNA used for PCR inhibition test after two times phenol–chloroform was a different extract from than the one used for tree times phenol–chloroform. The different volumes of DNA extracts from unspiked minced meat can thus be compared directly within each series from two times and three times phenol–chloroform extraction, respectively. An increase in Cq value was interpreted as PCR inhibition.

### PCR Assays

A real-time PCR assay was designed to detect and quantify Φ731 DNA by targeting the junction between *stx*_2a_ and *cat* using Primer3 (Untergasser et al., 2007). The resulting sequences were as follows: CGAAGTGATCTTCCGTCACA for the forward primer (StxCAT_F1), CCGCCATAAACATCTTCTTCA for the reverse primer (StxCAT_R2), and AGGAACTTCGGCGCGCCTAC for the FAM/BHQ1-labeled probe (StxCAT_probe1). Real-time PCR was performed with Brilliant III Ultra-Fast QPCR master mix (Agilent) with 900-nM primers and 200-nM probe on a CFX96 instrument (Bio-Rad) in a 20-μl reaction with 5-μl sample (approximately 10–100 ng DNA). Cycling conditions were 95°C for 3 min followed by 45 cycles of 5 s at 95°C and 10 s at 60°C. Uninoculated controls were analyzed by real-time PCR and were found to be negative.

Digital droplet PCR (ddPCR) was performed with ddPCR Supermix for Probes (Bio-Rad) and the same primer/probe combination as described above with 900-nM primers and 400-nM probe on a QX200 system (Bio-Rad) following the supplier's recommendations with 5-μl sample (approximately 10–100 ng DNA). PCR was performed on a T100 instrument (Bio-Rad) with 95°C for 10 min followed by 45 cycles of 30 s at 94°C, 1 min at 60°C, and ending with 98°C for 10 min. The ramp rate was 2°C/s.

### Bacteriological Analyses

Negative control samples to assess the general hygienic status in minced meat were analyzed quantitatively for *E. coli* and *Enterobacteriaceae*. The bacterial analyses were done at T0–1, for both experiments 1 and 2, T10 (experiment 1), and T20 (experiment 2), which were 2 and 1 day after use-by date, respectively. A total of 10 g minced meat was diluted in 0.9% peptone saline to obtain a 1:10 dilution. Serial dilutions were carried out in 0.9% peptone saline followed by plating on 3M Petrifilm™ Select E. coli Count Plate and Enterobacteriaceae Count Plate according the manufacturer's instructions (3M Health Care, St. Paul, MN, USA).

### Statistical Analysis

**Figure 2:**
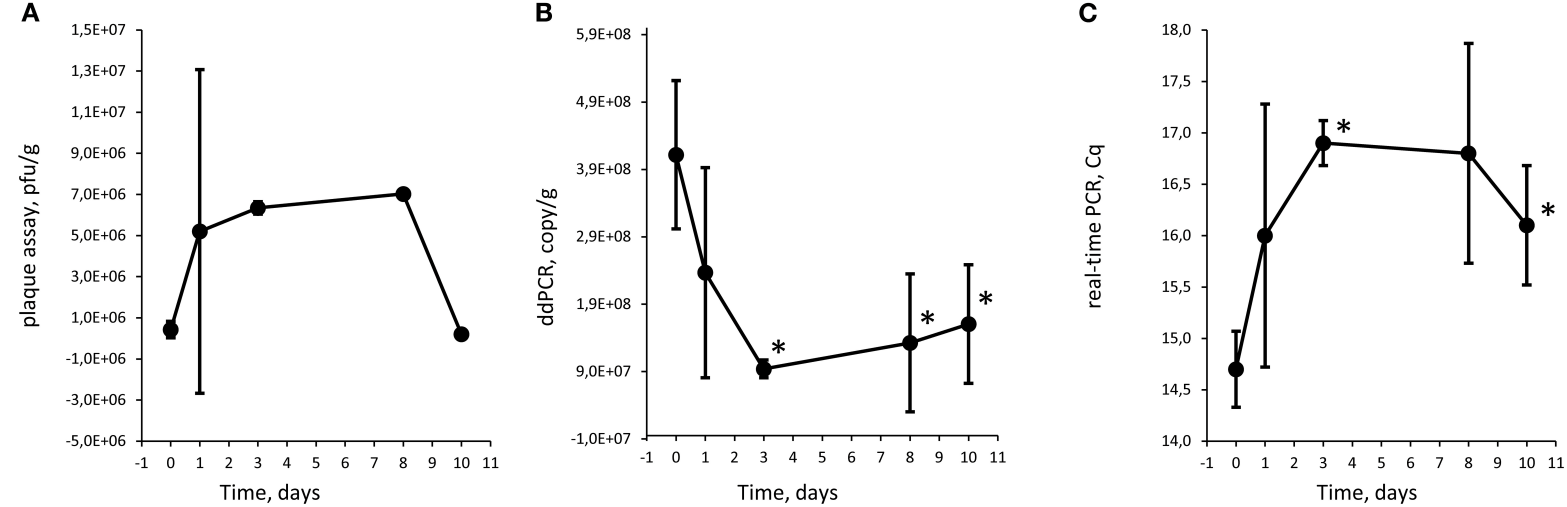
Quantification of Φ731 phages with plaque assay **(A)**, digital droplet PCR **(B)**, and detection with real-time PCR **(C)** as function of storage time at 8°C in experiment 1. Each value is the average of tree biological replicates ± SD. *Time points with a statistically significant decrease from T0 for digital droplet PCR and real-time PCR (*T*-test with *p* < 0.05). The Cq values were converted to values on a linear scale before statistical testing. The minced meat was inoculated with 3.1 × 10^5^ pfu/g.

**Figure 3:**
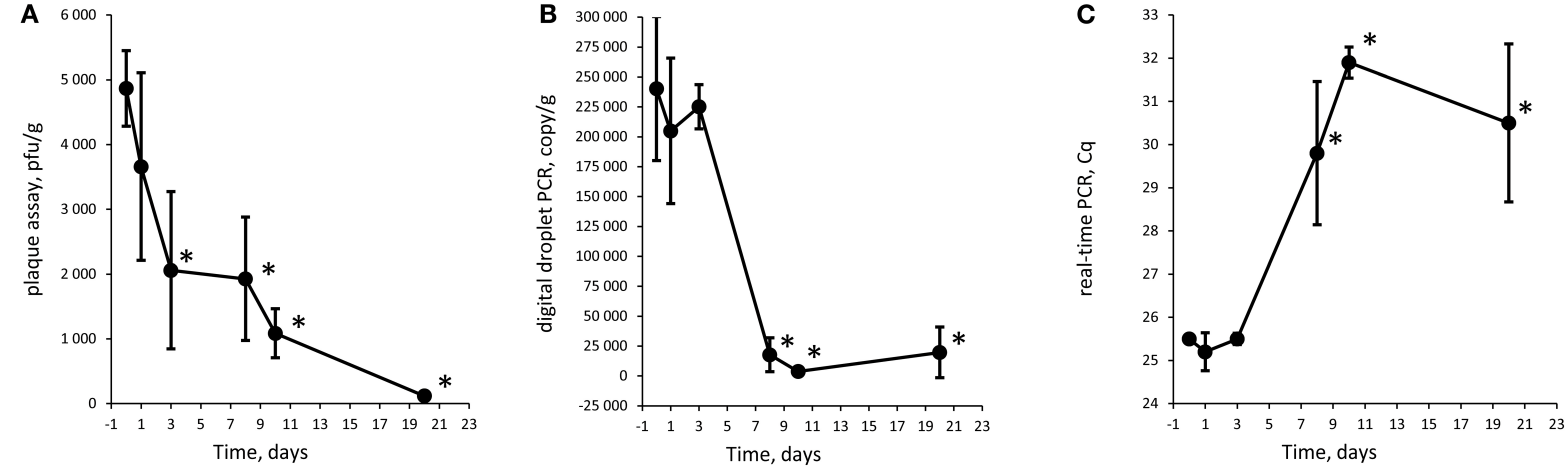
Quantification of Φ731 phages with plaque assay **(A)**, digital droplet PCR **(B)**, and detection with real-time PCR **(C)** as function of storage time at 8°C in experiment 2 (low spike). Each value is the average of tree biological replicates ± SD. *Time points with a statistically significant decrease from T0 for digital droplet PCR and real-time PCR (*T*-test with *p* < 0.05). The Cq values were converted to values on a linear scale before statistical testing. The minced meat was inoculated with 4.6 × 10^3^ pfu/g.

**Figure 4:**
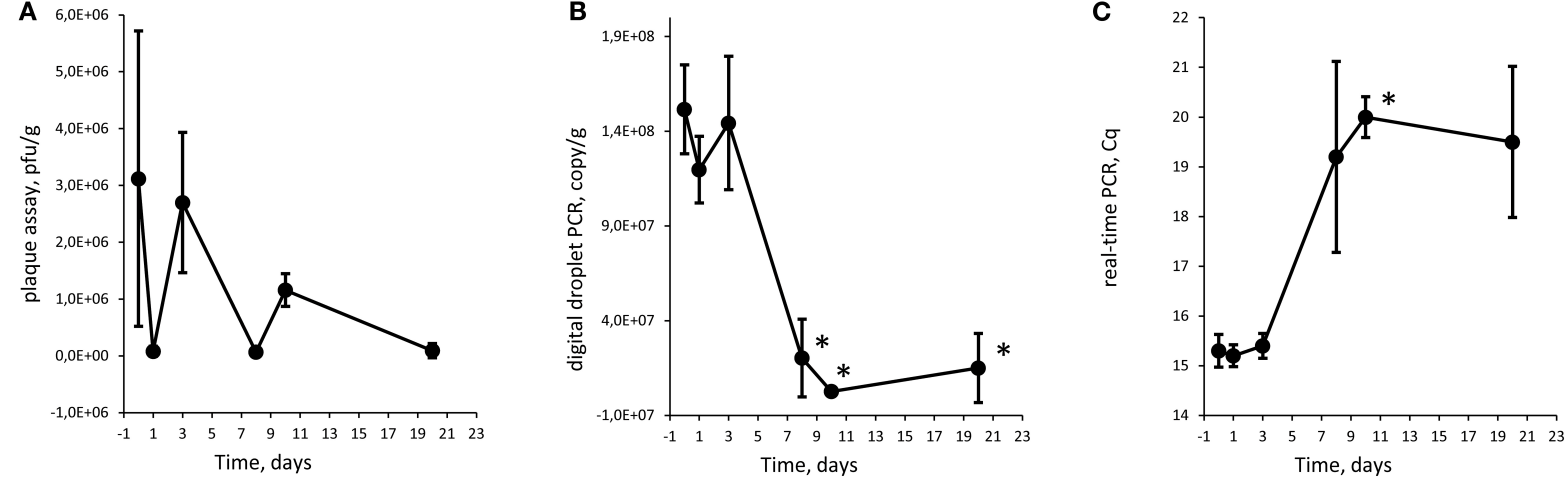
Quantification of Φ731 phages with plaque assay **(A)**, digital droplet PCR **(B)**, and detection with real-time PCR **(C)** as function of storage time at 8°C in experiment 2 (high spike). Each value is the average of tree biological replicates ± SD. *Time points with a statistically significant decrease from T0 for digital droplet PCR and real-time PCR (*T*-test with *p* < 0.05). The Cq values were converted to values on a linear scale before statistical testing. The minced meat was inoculated with 4.6 × 10^6^ pfu/g.

The result for each time point in experiments 1 and 2 was compared to T0 for their respective analysis method with a two-tailed *T*-test. The underlying data points are listed in Supplementary Tables 1–3. The Cq values were converted to an arbitrary value on a linear scale before statistical testing using the formula 2^Cq^ (Livak and Schmittgen, 2001). Several dilutions were analyzed in the plaque assays and with ddPCR. The dilutions were treated as technical replicates and averaged before calculating an average of the biological replicates. *p*-values < 0.05 were considered to be a significant decrease in numbers of phages or gene copies [marked with an asterisk (^*^) in Figures 2-4].

## Results and Discussion

### Pilot Study

Results from the plaque assays in the pilot experiment indicated that Φ731 could persist over time in the minced meat, albeit in decreasing numbers. Plaque assays were only performed on undiluted and one 10 fold dilution of phage filtrate resulting in full lysis and “too many plaques to count.” However, a slight reduction in plaque levels (results not shown) was observed during the period. The packages with minced meat were point inoculated through a silicone patch in order to maintain the modified atmosphere in the packs. When taking samples from a 400-g package for plaque assays and DNA extraction, it was difficult to ascertain that the 5 g used for analysis was taken from the spot of inoculation and that the inoculated material was included in the sample. These results suggested that we had sampled correctly and that the phages survived. The negative control samples revealed no native phages present that were able to infect the host strain used, indicating that *E. coli* C600 was an appropriate host strain. It was not possible to detect Φ731 by real-time PCR in this pilot experiment, most likely due to the DNA extraction method. The analyses of bacterial indicators were within the requirements in the European Microbiological Criteria (European Union, 2005) at T0 (results not shown). The experiences obtained in the pilot study were subsequently used in the following experiments.

### Modification of DNA Extraction

After analyzing the results from the pilot study employing the initial DNA extraction protocol, it was clear that modifications and optimizations were needed. The initial protocol used only a fraction of the phage filtrate (1 out of 10 ml) for DNA extraction and partly concentrated phages by PEG (see Supplementary Material 1 (1.1)]. However, this method was not optimized for isolation of phage DNA from minced meat.

Other options for the phage concentration step, as described by Jofre and Muniesa (2020), were tested [Supplementary Material 1 (1.3)]. Instead of using only parts of the phage filtrate as in the pilot study, we wanted to use the complete phage filtrate for DNA extraction. First, we tried to concentrate phages by centrifugal filters with molecular weight cutoff 100 KDa. However, this did not work optimally due to partly clogging of filters when applying this method on phage filtrate from minced meat [see Supplementary Material 1 (1.3)]. The final modification was using ultra-centrifugation for concentrating phages from the complete phage filtrate from each sample [Supplementary Material 1 (1.6) and Supplementary Material 2].

Other modifications were also implemented such as changing from the Wizard DNA Clean-Up System to phenol–chloroform extraction followed by precipitating DNA with 0.7 volume isopropanol (details in Supplementary Material 1 (1.2, 1.3)]. DNA extraction by phenol–chloroform is more time consuming but will give good results in terms of both DNA purity and the possibility to adjust the volume of buffer for resuspending the DNA pellet after alcohol precipitation. We resuspended the DNA pellet in a low volume of buffer to achieve more concentrated DNA.

The phenol–chloroform step was optimized by testing two and three times extractions [Supplementary Material 1 (1.6)]. DNA purity was assessed with a PCR inhibition test as described in section PCR Inhibition Test. No PCR inhibition could be observed at a standard PCR sample volume of ~5 μl regardless whether two or three times phenol–chloroform extraction was performed (Table 1). At a maximal sample volume (9.5 μl in a 20-μl PCR reaction), no significant PCR inhibition was observed when two times phenol–chloroform extraction was performed. Surprisingly, however, a significant PCR inhibition could be observed when three times phenol–chloroform extraction was performed (*T*-test, *p* < 0.05, Table 1). Thus, two times phenol–chloroform extraction was chosen. The high purity of the DNA extract gives freedom to increase the sample volume in the PCR analysis if samples with expected low phage concentration were to be analyzed.

**Table 1:** PCR inhibition test for DNA extraction of minced meat based on two and three times phenol–chloroform extraction.

**Volume of minced meat DNA (μl)**	**% minced meat DNA of reaction volume**	**Cq 2 × CHCl_**3**_ extraction**	**Cq 3 × CHCl_**3**_ extraction**
0	0	22.2	23.5
1.19	5.9	22.2	23.6
2.38	11.9	22.1	23.6
4.75	23.8	22.2	23.7
9.50	47.5	22.8	25.0^**^

*Increasing volumes of extracted DNA from minced meat were added to real-time PCR reactions with a constant amount of template for each condition (Φ731 phage DNA). The total reaction volume was 20 μl*.

*Each Cq value is the average of three biological replicates that were analyzed in two technical replicates each*.

** *p < 0.05 in a two-tailed Student T-test when compared to 0 μl minced meat DNA (3 × CHCl3 extraction). Each Cq value was converted to an arbitrary value on a linear scale before statistical analysis*.

Jofre and Muniesa (2020) described different methods for phage concentration and DNA extraction, and in the present study, all three concentration methods were tested with minor modifications. For the experiment presented here, ultra-centrifugation with the entire phage filtrate gave the highest yield for further DNA extraction. Together with two times phenol–chloroform extraction, we obtained DNA of sufficient quality for further PCR analyses. The optimized DNA extraction protocol is time-consuming and limited by the capacity of the ultra-centrifuge. This must, however, be balanced with the requirement of using an optimal method for obtaining reliable results.

### Design of Real-Time PCR Assay and Transfer to ddPCR

A real-time PCR assay was designed to detect the labeled Stx phage (Φ731) used. One of the junctions between the *stx* gene and the inserted chloramphenicol sequence was chosen as target. This assay has the potential to detect other Stx phages labeled with chloramphenicol by inactivating the *stx*_2a_ gene with the same strategy, since the *cat* gene will always be inserted in the same position (Serra-Moreno et al., 2006). The real-time PCR assay had an average amplification efficiency of 93.9% and linearity over six 10-fold dilutions calculated to *R*^2^ = 0.9995. Both values were well within our standard method performance acceptance values for amplification efficiency of 80–110% and linearity ≥0.98. Most real-time PCR assays can be transferred directly to ddPCR without extensive optimization. The designed assay was transferred to ddPCR with an increased probe concentration to the level recommended by the ddPCR instrument manufacturer with apparently acceptable performance.

Both real-time PCR and ddPCR have the capacity to quantify DNA. While real-time PCR depends on a pre-made standard curve that is needed to convert Cq values to DNA copies or DNA mass, ddPCR gives DNA copy numbers directly. ddPCR is reported to be slightly less sensitive to PCR inhibitors than real-time PCR (Rački et al., 2014; Zhao et al., 2016). In contrast to real-time PCR, partial PCR inhibition and variations in amplification efficiency do not affect quantification in ddPCR as ddPCR uses endpoint data and is therefore not dependent on amplification efficiency. Thus, ddPCR offers a reliable alternative to real-time PCR when quantitative analysis is needed and/or the samples are expected to contain PCR inhibitors.

### Experiments 1 and 2

To assess persistence of free phages over time, minced meat were spiked with Φ731 and the level of phages were measured at different time points. After the pilot study, it was decided to change from inoculating whole packages of minced meat to inoculating smaller portions in 50-ml tubes. This was done to enable the use all of the spiked sample and all the remaining phage filtrate for DNA extraction and subsequent PCR analyses. In experiment 1, the minced meat contained 14% fat and was 12 days beyond use-by date at T20, while in experiment 2, the minced meat was 1 day beyond use-by date at T20 and had 5% fat content.

For quantifying bacteriophages, three different methods were employed. Plaque assays were used as it is the traditional way for estimating phage numbers and have the benefit of measuring infective phages. Real-time PCR were used as it is commonly used in pathogen detection. We chose to use real-time PCR semi-quantitatively and use the Cq values and a linear representation of the Cq values directly to assess whether there was a trend in the plaque levels over the experimental period, without quantifying. ddPCR is an upcoming technique that we included to explore its feasibility in phage detection and quantification. ddPCR does, in addition, have the benefit of being less sensitive to PCR inhibition.

The Φ731 Stx phage could be detected by all three methods throughout the experimental period in both experiments (Figures 2-4). These results indicated that the inoculated Stx phages persist for at least 20 days regardless of the number of phages inoculated, content of fat in the minced meat, or different use-by date. Our results are in line with others (Rode et al., 2011; Langsrud et al., 2014; Nyambe et al., 2016) who have observed that free Stx phages may persist and remain infective for a period of time in foods and environmental samples. Langsrud et al. (2014) detected infective Stx phages in marinated meat stored at 4°C after 14 days although in greatly reduced numbers. Rode et al. (2011) showed that Stx phages in minced meat from beef were able to remain infective after heat treatment at 43 and 50°C for up to 120 min, while they had disappeared after 30 min at 60°C. Nyambe et al. (2016) indicated that a Stx phage inoculated into bovine feces and slurry survived and remained infective for at least 30 days under representative Irish temperatures. The results from the previous studies together with the presented study indicate that Stx phages persist and remain infective in various foods and environments relevant for food production, while stored and/or processed under different temperatures and environmental conditions.

Plaque assay, real-time PCR, and ddPCR all showed a decrease in phage levels over the experimental period. Statistical significance was tested with a *T*-test comparing each time point with T0, and time points with *p* < 0.05 are labeled with an asterisk (^*^) in Figures 2-4. There is no obvious explanations for why a significant decrease can be observed at around T8 for all three experiments measured by the molecular methods, but most importantly, the phages seem to persist to T20. However, it can be speculated if there are biological factors present in the meat, e.g., proteases and DNases that will inactivate the phages and degrade the DNA. In both experiments 1 and 2, the PCR results show an apparent increase in the level of phages at T10 and T20, respectively. However, this increase was not statistically significant when comparing T3 against T10 and T10 against T20 for experiments 1 and 2, respectively.

There is an apparent discrepancy between the amount of spike measured with plaque assay and quantification with ddPCR. ddPCR measures ~100 times more copies than the plaque assay estimates infective phages. For experiment 1, an increase in numbers of plaques from the plaque assays was observed from T0 to T1. The numbers of plaques remained stable for time points T3 and T8 before a decrease was observed at T10. It is not likely that the increase in plaques from T0 to T1 was due to native phages in the samples as the negative control samples revealed no plaques. Thus, this might be due to an analytical error during the set-up of the plaque assay. It could also be speculated if the ddPCR also picks up inactivated bacteriophages that will not infect the bacterial host and give countable plaques in the plaque assay. Plaque assays are sensitive to experimental conditions and host bacteria and are not necessarily able to measure all infective phages (Anderson et al., 2011). Thus, it has been proposed to use a corrections factor to correlate PCR results to plaque assay results (Anderson et al., 2011) as a discrepancy between plaque assay and PCR assays is not uncommon (Gentilomo et al., 2008). However, this has not been looked into in the present study.

When phage filtrate collected at T10 and T20 in experiments 1 and 2, respectively, was enriched with the host strain under lab conditions, the results indicated that the phages were able to form lysogens. A selection of potential lysogens was confirmed using the Φ731-specific real-time PCR. Plaque assays from non-inoculated control minced meat samples using *E. coli* C600 as a host did not reveal any presence of background phages that were able to infect the host, strongly suggesting that the phages detected were indeed the inoculated Φ731. Imamovic and Muniesa (2011) detected infective Stx phages in samples of minced meat after propagation of phages in host strains. However, these samples were obtained from supermarkets with phages naturally present, while our results are from inoculation experiments. This suggests that Stx phages have the ability to form lysogens after being kept at low temperatures over a period of time and then being subjected to apparently more favorable conditions for forming lysogens (i.e., 37°C). As previous Stx phage studies have indicated that transduction of phages will usually not occur at lower temperatures (Imamovic et al., 2009; Picozzi et al., 2012), there is a low risk of transduction events in storage of minced meat at 8°C. If the temperature during storage of food is kept low (4°C), free bacteriophages in food does not necessarily represent a large potential of forming new variants of STEC or other Shiga toxin-producing *Enterobacteriaceae* in the actual food product. However, there is a potential risk when Stx phages persist and remain infective in foods at low temperatures that transduction events with subsequent formation of new STEC variants might take place in foods at higher temperatures. Moreover, it facilitates transmission of Stx phages to humans and the possibility of transduction event occur in the gastrointestinal tract of humans where the temperature is optimal. It is thus important to obtain more knowledge of the persistence and infectivity of Stx phages in foods.

Results from the analyses for *E. coli* and *Enterobacteriaceae* are detailed in Table 2, indicating a very low number of *E. coli* present and within the European Microbiological Criteria (European Union, 2005), throughout the storage period at suboptimal temperature (8°C). The numbers of *Enterobacteriaceae* varied between the two experiments as expected since two different batches of minced meat were used and they were at different stages in their shelf life when the experiments took place. In experiment 1, an increase in number of *Enterobacteriaceae* was observed between T0–1 and T10, which is also not unexpected as the experiment started when the minced meat was in the middle of its storage period and the product was stored at suboptimal temperature.

**Table 2:** Results from the analyses of *E. coli* and *Enterobacteriaceae* in minced meat from beef.

	**Time**	***E. coli***	**Enterobacteriaceae**
Experiment 1	T0–1	<10	430
	T10	<10	5.3 × 10^5^
Experiment 2	T0-1	<10	10
	T20	<10	<10

*The analyses were carried out the day before inoculation with Φ731 (T0–1) and on day T10 (2 days after use-by date) in experiment 1 and T20 (1 day after use-by date) in experiment 2. The results are given as colony-forming units per gram*.

## Concluding Remarks

In the present study, we have used a modified and optimized DNA extraction method in combination with real-time PCR and ddPCR and plaque assays to study the persistence of a Stx phage in minced meat from beef stored at 8°C. The modified DNA extraction method includes ultra-centrifugation for concentration of phages prior to phenol–chloroform extraction. The ultra-centrifugation step allows the use of a larger volume of phage filtrate for DNA extraction. The subsequent steps in the DNA extraction result in flexibility in the amount of DNA available for use in PCR analyses, ultimately increasing the sensitivity of the method used for quantification of phages in a sample. The three quantification methods employed showed similar trends in the development of the phages during storage, where ddPCR have the benefit of giving absolute quantification of DNA copies in a simple experimental setup. The results indicate that the Stx phage persists and remains infective for at least 20 days under the storage conditions used in the present study. Stx phages in foods might represent a potential risk for humans. Although it can be speculated that transduction may take place at 8°C with subsequent forming of STEC, it can be expected to be a rare event. However, such an event may possibly take place under more optimal conditions, such as an increase in storage temperature of foods or in the gastrointestinal tract of humans.

## Data Availability Statement

The original contributions presented in the study are included in the article/Supplementary Material, further inquiries can be directed to the corresponding author.

## Author Contributions

GJ, AU, LN, and CS conceptualized the work. GJ, CS, and BS carried out the experiments, analyzed the data, and drafted the manuscript. All authors have contributed to the final version.

## Conflict of Interest

The authors declare that the research was conducted in the absence of any commercial or financial relationships that could be construed as a potential conflict of interest.
