# Supplementary material for: Persistence of a Stx-Encoding Bacteriophage in Minced Meat Investigated by Application of an Improved DNA Extraction Method and Digital Droplet PCR
Source: Front Microbiol. 2021 Jan 20;11:581575. doi: 10.3389/fmicb.2020.581575 (PMC7855172; doi:10.3389/fmicb.2020.581575)
Supplement: Supplementary file 1 [file Data_Sheet_1.docx]

SupplementaryMaterial 1

# Modifications of DNA extraction from pure phage filtrate and minced meat

We have tested several different modification steps for DNA extraction of phages. Some of the modifications were initially tested on pure phage filtrate before being tested on minced meat to evaluate whether the modifications increased the phage concentration and the DNA quality from the matrix.

## DNA extraction from miced meat in the pilot experiment

Sampling for the pilot experiment is described in 2.3.1 in the paper. The protocol was based on two protocols, one from Dr. Muniesa’s lab and a protocol from Hartzog & Ares, UC Santa Cruz Phage Genomics Lab. DNA extraction in the pilot experiment was performed as follows;

- - 1. 1 ml phage filtrate from minced meat was used for DNA extraction and
    2. Add 10 µl of DNase I (1U/µl, Promega) and 10 µl of RNaseA (20mg/ml, Sigma-Aldrich, cat.no R6513). Incubate at 37°C for 1 hour.
    3. Add 0.08 g NaCl and incubate on ice for 1 hour. Then, centrifuge at 10.000xg for 10 min.
    4. Transfer the supernatant to new tubes with 0.12 g PEG8000 (Sigma Life Science, BioUltra for molecular biology; 81268-250G). Dissolve everything and incubate for 1 hour on ice. Shake every 10^th^ minute. Spin for 45 min at 10.000xg.
    5. Discard the supernatant. Dissolve the pellet in 400 µl TE buffer.
    6. To purify the DNA go ahead with the Wizard DNA Clean-Up System (Promega, cat.no A7280).

The Wizard DNA Clean-Up system was chosen to avoid phenol-chloroform extractions. Real-time PCR was performed on the extracted phage DNA and all samples were negative when using 2 µl DNA, while using 5 µl DNA some of the samples were positive showing a high Cq. Also some of the control samples without the spike were slightly positive in the real-time PCR assay. For real-time PCR results, see Table A.

## Modifications of the DNA extraction from pure phage filtrate (step 1)

Testing triplicates for DNA extraction with different modifications;

1) Without adding DNase in in step 1.2.3 (below), phenol-chloroform extraction and precipitated with isopropanol (name of samples; 1-1 → 1-3)

2) Including step 1.2.3, phenol-chloroform extraction and precipitated with isopropanol (name of samples; 2-1 → 2-3);

3) Including step 1.2.3 and extracted DNA using the Wizard DNA Clean-Up System as described in 1.1.6 (name of samples; 3-1 → 3-3).

- - 1. Preparation of phage filtrate; 50 ml induced (C600::Φ731) is centrifuged at 4400 rpm in 15 min. The supernatant is filtered through 0.22 μm low protein binding membranes (large filters; Sterivex-GP, Millipore, cat no SVGPB1010).
    2. Phage filtrate was concentrated usingAmicon-Ultra-4 (Millipore) according to the manufacturer’s protocol and adjusting the volume of concentrated phage filtrate to 1 ml. All samples originated from 4 ml pure phage filtrate.
    3. Add 1 μl of DNase (1U/µl) and 1 μl of RNase (20mg/ml) and incubate at 37˚C for one hour.
    4. Add 0.08 g NaCl and incubate for one hour on ice. Centrifuge at 10.000xg in 10 min.
    5. Transfer the supernatant to new tubes and add 0.12 g PEG8000. Adjusted sample volume to 1 ml with SM buffer. Dissolve and incubate for 1 hour on ice and shake the tube every 10^th^ minute. Centrifuge at 10.000xg for 45 min. at 10.000xg.
    6. Air-dry the pellet and dissolve in 200 µl SM buffer. Add 67 µl Proteinase K buffer and 5 µl Proteinase K (20 mg/ml). Incubate at 50°C for one hour.
    7. DNA extraction using phenol-chloroform extraction (1x) was performed on samples; 1-1 → 1-3 and 2-1 → 2-3. The same volume of phenol-chloroform as sample volume were added to Phase Lock Gel tubes (light tubes; Quantabio) and centrifuged at max speed for 5 min.
    8. Precipitate with 0.7 volume isopropanol and spin max speed in 10 min at 0-4˚C.
    9. Recover the pellet in the bottom of the tube and wash with 1 ml 70% EtOH – spin max in 2 min.
    10. Let the pellet air-dry for one hour or more. Dissolve in 50 µl TE-buffer, pH 8.

#### Buffers:

**SM buffer:**

NaCl 2.9 g

MgSO_4_ x H_2_O 1 g

Tris HCl 1M, pH 7.5 25 ml

Gelatine 0.05 g

ddH_2_O up to 500 ml

**Proteinase K buffer:**

EDTA 0,5M, pH 8 2 ml

10% SDS 10 ml

Tris HCl 1M, pH 8 2 ml

ddH_2_O up to 100 ml

Large variation within triplicates of each modification step when measured in Nanodrop and based on real-time PCR. See table A. However, we will continue to use DNase in step 1.2.3, phenol-chloroform extraction and precipitation with isopropanol.

## Modifications of the DNA extraction from minced meat (step 1)

Testing different concentrations of phages on the following six samples:

1) Concentrated phage filtrate from minced meat; reduced from 4 ml to2 ml phage filtrate by using Amicon-Ultra-4 filters (name of samples; K1, K2, K3)

2) Concentrated phage filtrate from minced meat; reduced from 4 ml to 500 µl phage filtrate by using Amicon-Ultra-4 filters (name of samples; K1-500, K2-500, K3-500)

3) Non-concentrated phage filtrate from minced meat; using volume 8 ml directly (name of samples; K5, K6)

- - 1. Preparation of minced meat for DNA extraction
       1. 5 g minced meat, 100µl phage filtrate (concentration not known) and 15 ml PBS was added to two parallel samples.
       2. The samples were mixed in a Stomacher as described in 2.4.1 in the paper. Liquid samples from minced meat (approx. 8 ml) was centrifuged at 4400 rpm in 30 min. The supernatant is filtered through 0.22 μm low protein binding membranes.
    2. 4 ml filtrate from minced meat was concentrated using Amicon-Ultra-4 (Millipore) according to the manufacturer’s protocol.

*We aimed to concentrate 4 and 8 ml filtrate respectively, however after 40 min with centrifugation of 4 ml, 2 ml was still in the centrifugal filter. After 1hour 20 min approximately 500 µl filtrate was left.*

- - 1. The same protocol as described in 1.2.3-1.2.6 was used.
    2. DNA extraction using phenol-chloroform extraction (2x) as described in 1.2.7.
    3. Precipitate, wash, air-dry and dissolve as described in 1.2.8-1.2.10.

No detection of phage DNA in the real-time PCR assay. See table A. It might be too low concentration of phages and we might lose too much during the different steps.

## Modifications of the DNA extraction from minced meat (step 2)

Testing different concentrations of phages by using phage filtrate from minced meat directly without concentrating in Amicon-Ultra-4 filters:

1) 100 µl phage filtrate spiked in 5 g minced meat (name of samples; K1, K2)

2) 1 ml phage filtrate spiked in 5 g minced meat (name of samples; K100-1, K100-2)

- - 1. Preparation of minced meat for DNA extraction
       1. 5 g minced meat, phage filtrate (100 µl (6.3x10^6^ pfu) or 1 ml (6.3x10^7^ pfu)) and 15 ml PBS was added to two parallel samples.
       2. The samples were mixed in a Stomacher as described in “Preparation of Samples for DNA Extraction and Plaque Assays” in the paper. Liquid samples from minced meat (approx. 8 ml) was centrifuged at 4400 rpm in 30 min. The supernatant is filtered through 0.22 μm low protein binding membranes. After filtration, approximately 5 ml was remaining from each sample for the following steps.
    2. Add 2μl of DNase (1U/µl) and 2μl of RNase (20mg/ml) and incubate at 37˚C for one hour.
    3. Add 0.5 g NaCl and incubate for one hour on ice. Centrifuge at 10.000xg in 10 min.
    4. Transfer the supernatant to new tubes and add 0.8 g PEG8000. Dissolve and incubate for 1 hour on ice and shake the tube every 10^th^ minute. Centrifuge at 10.000xg for 45 min. at 10.000xg.
    5. Air-dry the pellet and dissolve in 2 ml SM buffer. Add 1 ml Proteinase K buffer and15 µl Proteinase K (20 mg/ml). Incubate at 50°C for one hour.

*With this large volume, we needed two tubes for the following phenol-chloroform extraction.*

- - 1. DNA extraction using phenol-chloroform extraction (2x) as described in 1.2.7.
    2. Precipitate, wash, air-dry and dissolve as described in 1.2.8-1.2.10.

No detection of phage DNA in the real-time PCR assay. See table A. Might be too low concentration of phages and we might lose too much during the different steps.

## Modifications of the DNA extraction from pure phage filtrate (step 2)

Testing ultra-centrifugation for concentration of phages in pure phage filtrate and testing the difference between 2x and 3x phenol-chloroform extraction;

1) 2x phenol-chloroform extraction (name of samples; Φ731-2-1, Φ731-2-2, Φ731-2-3);

2) 3x phenol-chloroform extraction (name of samples; Φ731-3-1, Φ731-3-2, Φ731-3-3)

- - 1. Preparation of phage filtrate as described in 1.2 but at a 200 ml volume. Large sterile filters (Steritop 0.22µM, GP Millipore Express, Cat.no: SCGPT01RE). This phage filtrate was used for plaque assay for quantification of bacteriophages and DNA extraction with concentration of bacteriophages using ultra-centrifugation.
    2. Ultra-centrifugation using rotor SW 41 TI swinging-bucket at 100.000 x g (Beckman Coulter, Life Science) in two hours using tubes with approximately 11 ml volume (Ultra-Clear tubes 14x89 mm, Cat.no: 344059). All tubes (six in total) were filled with phage filtrate and treated as one sample.
    3. Remove the supernatant and dissolve the pellet in 200 µl SM Buffer.
    4. Add 1 μl DNase (1U/µl, Promega) and 1 μl RNaseA (20mg/ml, Sigma-Aldrich) and incubate for one hour at 37˚C.
    5. Add 67 µl Proteinase K buffer and 5 µl Proteinase K (20 mg/ml, Qiagen). Incubate at 50°C for one hour.
    6. DNA extraction using phenol-chloroform extraction as described in 1.2.7.
    7. Precipitate, wash, air-dry and dissolve as described in 1.2.8-1.2.10.

The quality of DNA seems to be of high quality regardless two or three times phenol-chloroform extraction. The DNA quantity seems to be higher in general in three times phenol-chloroform than two times phenol-chloroform extraction. See table A.

## Modifications of the DNA extraction from minced meat without spiking of phages (step 3)

Testing ultra-centrifugation for minced meat samples (without spiking of phage Φ731) and testing the difference between 2x and 3x phenol-chloroform extraction;

1) 2x phenol-chloroform extraction (name of samples; K1, K2, K3);

2) 3x phenol-chloroform extraction (name of samples; K4, K5, K6)

- - 1. Preparation of minced meat for DNA extraction
       1. 10 g minced meat and 30 ml PBS was added for three samples in parallel.
       2. The samples were mixed in a Stomacher as described in 2.4.1 in the paper. As much liquid as possible per sample was transferred and mixed in 50 ml Falcon tubes and centrifuged at v/10.000xg in 30 min. In total, we had approximately 45 ml liquid per triplicates.
       3. The mixed samples were filtered through a large filter as describe in 1.5.1.
    2. The samples were divided equally into three tubes each for ultra-centrifugation and centrifugation was performed as described in 1.5.2.
    3. The following steps were performed as described in 1.5.3-1.5.5.
    4. DNA extraction using phenol-chloroform extraction as described in 1.2.7.
    5. Precipitate, wash, air-dry and dissolve as described in 1.2.8-1.2.10.

Very low concentration of phage DNA as expected since no phages were spiked in. This DNA from unspiked minced meat were used in a PCR inhibition test described in the paper.

**Table A**. Nanodrop results (nucleic acid concentration, A260/A280, A260/A2230) and real-time PCR results (different volumes tested for different experiment, and values from real-time PCR are given in Cq) shown for each of the DNA extraction modifications performed. Real-time PCR applied is the one described in 2.6 in the paper. Experiment refers to the number the specific experiment is given in this supplementary file.

|  |  | **Nanodrop** | | | **Real-time PCR** | | |
| --- | --- | --- | --- | --- | --- | --- | --- |
| **Exp.** | **Name of sample** | **Nucleic acid conc. (ng(µl)** | **A260/280** | **A260/230** | **Cq**  **(2µl DNA)** | **Cq**  **(2µl DNA (1:10 dilution))** | **Cq**  **(5 µl DNA)** |
| 1.1. Pilot project | KB0-1 | - | - | - | No Cq | - | 34.0 |
|  | KB0-2 | - | - | - | No Cq | - | No Cq |
|  | KB0-3 | - | - | - | No Cq | - | No Cq |
|  | KB1-1 | - | - | - | No Cq | - | No Cq |
|  | KB1-2 | - | - | - | No Cq | - | No Cq |
|  | KB1-3 | - | - | - | No Cq | - | No Cq |
|  | KB3-1 | - | - | - | No Cq | - | 33.9 |
|  | KB3-2 | - | - | - | No Cq | - | No Cq |
|  | KB3-3 | - | - | - | No Cq | - | No Cq |
|  | KF0-1 | - | - | - | No Cq | - | No Cq |
|  | KF0-2 | - | - | - | No Cq | - | No Cq |
|  | KF0-3 | - | - | - | No Cq | - | No Cq |
|  | KF1-1 | - | - | - | No Cq | - | No Cq |
|  | KF1-2 | - | - | - | No Cq | - | No Cq |
|  | KF1-3 | - | - | - | 32.4 | - | 28.9 |
|  | KF3-1 | - | - | - | No Cq | - | No Cq |
|  | KF3-2 | - | - | - | No Cq | - | 32.0 |
|  | KF3-3 | - | - | - | No Cq | - | 33.2 |
| 1.2 | 1-1 | 195.9 | 1.85 | 2.34 | 11.5 | 11.1 | - |
|  | 1-2 | 1283 | 1.88 | 2.02 | 10.4 | 14.7 | - |
|  | 1-3 | 163.9 | 1.86 | 2.31 | No Cq | 9.4 | - |
|  | 2-1 | 513.7 | 1.9 | 2.38 | 9.6 | 14.4 | - |
|  | 2-2 | 1575.8 | 1.87 | 2.07 | 9.8 | 8.3 | - |
|  | 2-3 | 96.7 | 1.85 | 2.47 | 9.4 | 8.7 | - |
|  | 3-1 | 169.7 | 1.77 | 0.25 | 8.3 | 11.1 | - |
|  | 3-2 | 134.5 | 1.79 | 0.17 | 9.7 | 12.0 | - |
|  | 3-3 | 173.8 | 1.75 | 0.24 | 9.3 | 11.2 | - |
| 1.3 | K1 | 26.5 | - | - | - | - | No Cq |
|  | K2 | 18.9 | - | - | - | - | No Cq |
|  | K3 | 20.8 | - | - | - | - | No Cq |
|  | K1-500 | 11.5 | - | - | - | - | No Cq |
|  | K2-500 | 13.3 | - | - | - | - | No Cq |
|  | K3-500 | 10.3 | - | - | - | - | No Cq |
|  | K5 | 33.3 | - | - | - | - | No Cq |
|  | K6 | 47.4 | - | - | - | - | No Cq |
| 1.4 | K1-1 | 121.1 | 1.45 | 0.52 | No Cq | - | - |
|  | K1-2 | 134.8 | 1.46 | 0.51 | No Cq | - | - |
|  | K100-1 | 83.2 | 1.38 | 0.43 | No Cq | - | - |
|  | K100-2 | 74.6 | 1.38 | 0.43 | No Cq | - | - |
| 1.5 | Φ731-2-1 | 308.2 | 1.85 | 1.92 | - | - | - |
|  | Φ731-2-2 | 249.3 | 1.85 | 1.92 | - | - | - |
|  | Φ731-2-3 | 1438.7 | 1.87 | 1.83 | - | - | - |
|  | Φ731-3-1 | 471.6 | 1.8 | 1.99 | - | - | - |
|  | Φ731-3-2 | 437.5 | 1.83 | 1.92 | - | - | - |
|  | Φ731-3-3 | 469.1 | 1.8 | 1.91 | - | - | - |
| 1.6 | K1 | 0.43 | -0.52 | 0.57 | - | - | - |
|  | K2 | 0.79 | 1.06 | 0.58 | - | - | - |
|  | K3 | 0.88 | 0.84 | 0.61 | - | - | - |
|  | K4 | 4.17 | 1.4 | 0.9 | - | - | - |
|  | K5 | 2.03 | 4.3 | 0.78 | - | - | - |
|  | K6 | 5.62 | 1.36 | 0.75 | - | - | - |
