# Supplementary material for: Persistence of a Stx-Encoding Bacteriophage in Minced Meat Investigated by Application of an Improved DNA Extraction Method and Digital Droplet PCR
Source: Front Microbiol. 2021 Jan 20;11:581575. doi: 10.3389/fmicb.2020.581575 (PMC7855172; doi:10.3389/fmicb.2020.581575)
Supplement: Supplementary file 2 [file Data_Sheet_2.docx]

Supplementary material 2

# Detailed DNA extraction protocol for bacteriophages from minced meat

This protocol include a description of DNA extraction from sample to phage filtrate to ultra-centrifugation to phenol-chloroform extraction.

1. Transfer the sample to a Stomacher bag with filter (VWR® Blender bag, Lateral filter, 400 ml, Sterile) and dilute the sample in PBS in 1:4, followed by homogenization in a Stomacher for 1 min.
2. Collect as much liquid as possible from the samples and transfer to 50 ml tubes before centrifuging at 10 000 x g for 30 min at 4°C (Multifuge X3R, Thermo Scientific).
3. The supernatant is filtrated through a 0.22 µm filter (Steritop 0.22 µm, 150 ml, Millipore, Merck, Darmstadt, Germany).
4. Ultra-centrifugation
   1. Transfer phage filtrate to tubes for ultra-centrifugation (Ultra-Clear tubes 14x89 mm, Cat.no: 344059, Beckman) up to 13 ml phage filtrate per tube.
   2. Ultra-centrifugation using rotor SW 41 TI swinging-bucket at 100.000 xg (35.000 rpm) in two hours.
5. Remove the supernatant and dissolve the pellet in 200 µl SM Buffer.
6. Add 1 μl DNase (1u/µl, Promega) and 1 μl RNaseA (20mg/ml, Sigma-Aldrich) and incubate for one hour at 37˚C.
7. Add 67 µl Proteinase K buffer and 5 µl Proteinase K (20 mg/ml, Qiagen). Incubate at 50°C for one hour.
8. Phenol-chloroform extraction
   1. Use Phase Lock gel tubes (PLG, Quantabio) for phenol-chloroform extraction. Before use spin empty PLG tubes at 12-16.000 x g in 20-30 sec. Then, divide the sample into two tubes and add the same volume of phenol-chloroform (Phenol:Chloroform:Isoamyl Alcohol 25:24:1, Sigma-Aldrich), mix gently to a homogeneous solution, but do not vortex.
   2. Spin at maximum speed (12.000-16-000 x g) in five min to separate the phases
   3. Remove carefully the upper phase (water phase) and repeat the phenol-chloroform extraction
9. Precipitate DNA with 0.7 volume isopropanol
   1. Add 0.7 volume isopropanol to the sample.
   2. Centrifuge at maximum speed in 10 min at 0-4˚C.
   3. Remove supernatant and wash the pellet with 1 ml 70% ethanol.
   4. Centrifuge at maximum speed in 2 min.
10. Remove supernatant and let the pellet air dry overnight.
11. Resuspend the pellet in 50 µl TE-buffer, pH 8.0.

### Buffers:

**SM buffer:**

NaCl 2.9 g

MgSO_4_ x H_2_O 1 g

Tris HCl 1M, pH 7.5 25 ml

Gelatine 0.05 g

ddH_2_O up to 500 ml

**Proteinase K buffer:**

EDTA 0.5M, pH 8.0 2 ml

10% SDS 10 ml

Tris HCl 1M, pH 8.0 2 ml

ddH_2_O up to 100 ml
