# Supplementary material for: Persistence of a Stx-Encoding Bacteriophage in Minced Meat Investigated by Application of an Improved DNA Extraction Method and Digital Droplet PCR
Source: Front Microbiol. 2021 Jan 20;11:581575. doi: 10.3389/fmicb.2020.581575 (PMC7855172; doi:10.3389/fmicb.2020.581575)
Supplement: Supplementary file 3 [file Table_1.docx]

Supplementary table 1. Raw data from experiment 1 plotted in figure 2. For each time-point were three biological replicates taken and analyzed. Each time-point is shown as mean ± SD.

| Time, days | Acronym | Plaque assay, plaque/g ± SD |  | ddPCR, cp/g ± SD | T-test*, p | rtPCR, Cq ± SD | rtPCR, linearized value** ± SD | T-test*, p |
| --- | --- | --- | --- | --- | --- | --- | --- | --- |
| 0 | T0 | 426 000 ± 69 541 |  | 20 590 431 ± 5 496 115 | - | 14.7 ± 0.37 | 27 004 ± 7 279 | - |
| 1 | T1 | 5 200 000 ± 400 000 |  | 11 842 327 ± 7 791 944 | NS | 16.0 ± 1.28 | 87 506 ± 83 098 | NS |
| 3 | T3 | 6 346 666 ± 7 864 892 |  | 4 698 789 ± 659 584 | < 0.05 | 16.9 ± 0.22 | 126 372 ± 18 750 | < 0.05 |
| 8 | T8 | 7 026 666 ± 300 222 |  | 6 633 076 ± 5 109 630 | < 0.05 | 16.8 ± 1.07 | 130 001 ± 72 073 | NS |
| 10 | T10 | 201 333 ± 189 371 |  | 8 030 286 ± 4 401 482 | < 0.05 | 16.1 ± 0.58 | 73 637 ± 26 226 | < 0.05 |

* A two tailed T-test was used to assess whether there was a statistical significant decrease compared to T0, NS = not significant.

** The Cq value were converted to an a value proportional to the initial DNA concentration on linear scale with the formula 2^Cq^
