# Supplementary material for: Persistence of a Stx-Encoding Bacteriophage in Minced Meat Investigated by Application of an Improved DNA Extraction Method and Digital Droplet PCR
Source: Front Microbiol. 2021 Jan 20;11:581575. doi: 10.3389/fmicb.2020.581575 (PMC7855172; doi:10.3389/fmicb.2020.581575)
Supplement: Supplementary file 4 [file Table_2.docx]

Supplementary table 2. Raw data from experiment 2 with low spike plotted in figure 3. For each time-point were three biological replicates taken and analyzed. Each time-point is shown as mean ± SD.

| Time, days | Acronym | Plaque assay, plaque/g ± SD | T-test*, p | ddPCR, cp/g ± SD | T-test*, p | rtPCR, Cq ± SD | rtPCR, linearized value** ± SD | T-test*, p |
| --- | --- | --- | --- | --- | --- | --- | --- | --- |
| 0 | T0 | 4 867 ± 583 | - | 240 387 ± 60 195 | - | 25.5 ± 0.05 | 45 961 332 ± 1 597 773 | - |
| 1 | T1 | 3 660 ± 1 448 | NS | 204 893 ± 60 837 | NS | 25.2 ± 0.44 | 39 696 958 ± 11 219 737 | NS |
| 3 | T3 | 2 060 ± 1 215 | < 0.05 | 225 207 ± 18 499 | NS | 25.5 ± 0.13 | 47 365 158 ± 4 272 514 | NS |
| 8 | T8 | 1 927 ± 952 | < 0.05 | 17 769 ± 14 201 | < 0.05 | 29.8 ± 1.66 | 1 507 576 319 ± 1 720 902 296 | < 0.05 |
| 10 | T10 | 1 087 ± 378 | < 0.05 | 3 879 ± 760 | < 0.05 | 31.9 ± 0.36 | 4 060 984 806 ± 9 45 780 611 | < 0.05 |
| 20 | T20 | 120 ± 0 | < 0.05 | 1 9734 ± 21 181 | < 0.05 | 30.5 ± 1.83 | 2 244 811 090 ± 1 703 969 488 | < 0.05 |

* A two tailed T-test was used to assess whether there was a statistical significant decrease compared to T0, NS = not significant

** The Cq value were converted to an a value proportional to the initial DNA concentration on linear scale with the formula 2^Cq^
