# Supplementary material for: Persistence of a Stx-Encoding Bacteriophage in Minced Meat Investigated by Application of an Improved DNA Extraction Method and Digital Droplet PCR
Source: Front Microbiol. 2021 Jan 20;11:581575. doi: 10.3389/fmicb.2020.581575 (PMC7855172; doi:10.3389/fmicb.2020.581575)
Supplement: Supplementary file 5 [file Table_3.docx]

Supplementary table 3. Raw data from experiment 2 with high spike plotted in figure 4. For each time-point were three biological replicates taken and analyzed. Each time-point is shown as mean ± SD.

| Time, days | Acronym | Plaque assay,  plaque/g ± SD | T-test*, p | ddPCR, cp/g ± SD | T-test*, p | rtPCR, Cq ± SD | rtPCR, linearized value** ± SD | T-test*, p |
| --- | --- | --- | --- | --- | --- | --- | --- | --- |
| 0 | T0 | 3 120 000 ± 2 598 076 | - | 151 555 555 ± 23 459 944 | - | 15.3 ± 0.33 | 41 310 ± 9 134 | - |
| 1 | T1 | 81 333 ± 88 934 | NS | 11 748 444 ± 17 646 781 | NS | 15.2 ± 0.22 | 37 306 ± 5 340 | NS |
| 3 | T3 | 2 700 000 ± 1 233 045 | NS | 144 334 667 ± 35 223 371 | NS | 15.4 ± 0.25 | 43 898 ± 7 965 | NS |
| 8 | T8 | 68 000 ± 74 081 | NS | 20 341 689 ± 20 531 382 | < 0.05 | 19.2 ± 1.92 | 1 044 855 ± 1 148 038 | < 0.05 |
| 10 | T10 | 1 160 000 ± 288 444 | NS | 2 677 645 ± 303 902 | < 0.05 | 20.0 ± 0.41 | 1 095 877 ± 28 4327 | < 0.05 |
| 20 | T20 | 93 333 ± 127 027 | NS | 15 053 378 ± 18 290 360 | < 0.05 | 19.5 ± 1.52 | 1 011 266 ± 716 485 | < 0.05 |

* A two tailed T-test was used to assess whether there was a statistical significant decrease compared to T0, NS = not significant

** The Cq value were converted to an a value proportional to the initial DNA concentration on linear scale with the formula 2^Cq^
